# “Trees Live on Soil and Sunshine!”- Coexistence of Scientific and Alternative Conception of Tree Assimilation

**DOI:** 10.1371/journal.pone.0147802

**Published:** 2016-01-25

**Authors:** Christine Johanna Thorn, Kerstin Bissinger, Simon Thorn, Franz Xaver Bogner

**Affiliations:** 1 Department of Biological Education and Centre of Math and Science Education (Z-MNU), University of Bayreuth, Universitätstraße 30, NW II, 95447, Bayreuth, Germany; 2 Bavarian Forest National Park, National Park, Freyunger Str. 2, 94481, Grafenau, Germany; University of Catania, ITALY

## Abstract

Successful learning is the integration of new knowledge into existing schemes, leading to an integrated and correct scientific conception. By contrast, the co-existence of scientific and alternative conceptions may indicate a fragmented knowledge profile. Every learner is unique and thus carries an individual set of preconceptions before classroom engagement due to prior experiences. Hence, instructors and teachers have to consider the heterogeneous knowledge profiles of their class when teaching. However, determinants of fragmented knowledge profiles are not well understood yet, which may hamper a development of adapted teaching schemes. We used a questionnaire-based approach to assess conceptual knowledge of tree assimilation and wood synthesis surveying 885 students of four educational levels: 6^th^ graders, 10^th^ graders, natural science freshmen and other academic studies freshmen. We analysed the influence of learner’s characteristics such as educational level, age and sex on the coexistence of scientific and alternative conceptions. Within all subsamples well-known alternative conceptions regarding tree assimilation and wood synthesis coexisted with correct scientific ones. For example, students describe trees to be living on “soil and sunshine”, representing scientific knowledge of photosynthesis mingled with an alternative conception of trees eating like animals. Fragmented knowledge profiles occurred in all subsamples, but our models showed that improved education and age foster knowledge integration. Sex had almost no influence on the existing scientific conceptions and evolution of knowledge integration. Consequently, complex biological issues such as tree assimilation and wood synthesis need specific support e.g. through repeated learning units in class- and seminar-rooms in order to help especially young students to handle and overcome common alternative conceptions and appropriately integrate scientific conceptions into their knowledge profile.

## Introduction

Since the beginning of the last century, scientists have been interested in the organisation of cognitive knowledge. Piaget [1] already explained intelligence to be organising the world by organising itself. Thus, new knowledge schemes (organized patterns of knowledge that arrange categories of information and relationships among them) could be developed by modifying old ones [2]. Consequently, successful learning is understood beyond the rote memorisation of mere facts as being the integration of new knowledge into existing schemes. This basic interpretation of daily knowledge acquisition is limited in classrooms to which students bring robust, pre-existing conceptions differing from the accepted scientific ones [3]. Therefore instructors face fragmented to well-structured knowledge profiles which vary between individual students according to prior experiences [4]. Structuring knowledge is important as an individual’s conceptual knowledge consists of various elements such as observations, beliefs, explanations etc. [5,6]. These elements are relevant in the process by which fragmentation and integration contribute to a person’s conceptual knowledge [7]. Knowledge fragmentation potentially leads to coexisting parallel conceptions related to specific settings (e.g. social environment) [2,7–9].

Different scientific disciplines argue for two parallel assumptions about students´ conceptions: The psychological approach of Schneider and Hardy [7] comprises three conceptions namely misconception, every-day and scientific conception. Misconception and everyday conception are both alternative conceptions, which can be falsified by scientific experiments. Scientific conceptions relate to the current state of scientific knowledge, which can be verified but not falsified through an experiment. Misconceptions and everyday conceptions vary in their explanatory power: while everyday conceptions coherently explain observations from everyday life; misconceptions imply no explanatory power and thus can be reduced to naïve concepts [7]. However, in contemporary science education and in scientific literature, the word “misconception” was found to be rarely and inconsistently used even leading to the statement: “Misconceptions are so yesterday” [3] (p. 352). In the 1980s and 1990s when researchers frequently analysed students’ conceptions in different fields, the term “misconception” was commonly used to describe frequent scientifically incorrect conceptions that demand professional instruction to be overcome and replaced [10].

Authors such as Hammer [11] introduced students’ naïve ideas as valuable resources for developing more sophisticated scientific understanding in physics; supporting Smith et al. [12], who argued that misconceptions contradict constructivism that provoke a paradigm shift. Thus the term “misconception”, which historically was aligned with eradication and or replacement of conceptions, should not be used in biology education research any more [3]. The term “alternative conception” seems appropriate as it refers to “experience-based explanations constructed by a learner to make a range of natural phenomena and objects intelligible” while conferring “intellectual respect on the learner who holds those ideas” [13] (p.56). Consequently, we focus on the currently accepted second approach: separating students’ knowledge into scientific and alternative conceptions.

Recent studies on biological conceptions predominantly investigate the understanding of evolution and natural selection [14–19]. Conceptual studies on photosynthesis and related issues such as tree assimilation and wood synthesis date back to the 1980s, revealing one prominent alternative concept: Plants absorb nutrients from their environment [20,21]. In detail, fifty percent of participating ninth graders in an Israeli study dealing with photosynthesis thought that trees absorb nutrients from the environment [21] and more than one third of surveyed German students assumed even that plants absorb sugar from soil [22], neglecting the role of plants as primary producers. Hence, students often do not understand plants as autotrophic organisms [22] that convert gas (CO_2_) to plant biomass [21]. Although the existence of alternative conceptions of tree assimilation and wood synthesis is proven, determinants of the coexistence of scientific and alternative conceptions remain unclear.

We used questionnaires comprising one basic question that focused on enumerating factors assimilated by trees and a more complex question that required a deeper understanding of the wood synthesis process. We investigated potential explanatory factors (age, sex and educational background) on (I), the *expression* of scientific and alternative conceptions and (II) the *coexistence* of scientific and alternative conceptions.

## Methods

### Ethics statement

All proposed research and consent processes were approved by the Bavarian Ministry of Education (“Bayerisches Staatsministerium für Bildung und Kultus, Wissenschaft und Kunst”) in October 2013 (II.7–5 O 5106/92/7) and in November 2013 (III.9–5 O 5106/91/13). All principals of the participating schools were informed about the study and the research conducted in their classrooms and provided their consent. All participants provided their written consent to participate in this study. Students who had not reached age of consent also provided the written consent of their legal guardians. Prior to the data collection, the purpose of the study was explained to all participants. Data privacy laws were respected as our data was recorded pseudo anonymously. Each participant provided a specific identifier number, based on their sex, birth month and year, first two letters of their mothers name and house number. Any categorisation of sex is based on the self-reported sex according to the identifier number provided by the students within the questionnaire. The permit numbers of the Bavarian Ministry of Education allow public review of all questionnaires used in the study. All students and parents from participating classes had the chance to reject study participation, but no one exercised this right.

### Data collection

We gathered our data at two universities and five schools, located in the federal state Bavaria in Germany. Prior to the main study, we sampled responses of 113 freshmen (44.25% male, 55.75% female; mean age = 22.4±2.3) to develop valid test items. All students responded on two open questions focussing on conceptions of tree assimilation and subsequent wood synthesis. Since the way a question is posed might influence the answer, we conduct a pre-test-study to test different wordings and develop both questions. For instance, in question A: “[…] In your opinion, what does a tree assimilate in order to form a thick trunk?” Students answered ‘sugar’, which can be either a scientific correct concept if it corresponds to the production of starch and cellulose, or an alternative concept in terms of plants assimilating sugar from their environment. Hence, we reworded the original question and we added the description “from its environment” to clarify our intension. Altogether three test-runs were implemented to develop our final questions that do not allow ambiguous answers.

We used the following two items in the present study: A) “One of the oldest and thickest trees in Bavaria is a 600-year old oak with a circumference of 7.1 m. In your opinion, what does this tree assimilate from its environment during the day in order to form such a thick trunk?”, which represents a basic question and B) “Explain in detail how, in your opinion, this tree produces its timber with inclusion of the above mentioned terms.”, which is a more complex question aiming to reveal substantial understanding of the biological processes of tree assimilation and wood synthesis.

We used these open questions in order to avoid any restriction (possibly conveyed by closed- or multiple-choice questions) and for capturing all concepts provided by students. In total, 885 students (46.2% male; 53.8% female; mean age = 18.71 SD±3.87) participated in our two question paper-and-pencil questionnaire. A detailed sample description including demographic data is provided in S1 Table.

### Data analysis

Prior to statistical analysis we determined categories by applying a qualitative content analysis [23] to structure and condense our data by an inductive bottom-up approach. Reliability of category assignment was estimated by an intra- inter-rater design (Cohen´s Kappa) [24,25]. Thereby randomly chosen 10% of all answers given by participants were dedicated to the categories built: by the same person (intra-rater) and by another person (inter-rater), who was not familiar with the data before. The higher the agreement of dedicated categories is, the closer the reliability (Cohen´s Kappa = 0–1; 1 meaning 100% agreement). This procedure revealed 11 categories for each question respectively, which were assigned to scientific or alternative conceptions (S2 Table). Those conceptions were converted into binomial data, representing the presence or absence of a specific category in a student. The sum of present categories indicates the expression of scientific or alternative concepts in a student ranging depended on question A or B and number of concepts from zero to five or six (question A: five scientific and six alternative concepts, question B: six scientific and five alternative concepts) (S2 Table). All concepts encountered were assigned to either alternative or scientific conceptions. For definition we used two terms in this study: concept and conception. Concept refers to particular students’ ideas and conception reflects the nature of understanding (e.g. all collected ideas, which meant similar issues). For instance, a student’s answer such as “a tree eats soil” resulting in the conception defined as the abstract comprehension of something’s nature, in this case an alternative understanding of tree assimilation “nutrients taken from soil” (= category). We assigned “mineral(s)” (i.e. all inorganic substances that trees may absorb from soil), to scientific concepts and “nutrients” (i.e. long-chain hydrocarbons, fats and proteins) to alternative concepts, since trees are autotrophic organism that to not absorb nutrients from the environment.

All subsequent, analyses were conducted in R (The R Development Core Team 2014, version 3.1.1; www.r-project.org). To explore general coexistence of distinct concepts we fit Ward´s hierarchical cluster analysis [26] by means of function *hclust* (R-package *stats*). Afterwards we implemented k-means cluster analysis [27] by means of function *k-mean* (R-package *stats*) to analyse the structure of the determined clusters. The approach was validated by means of a contingency table [28]. Coefficient of contingency (*C*) describes the interrelation between two variables and is always 0 < C < 1, whereas high *C* means high relations (highest accessible *C* = *C*_max_).

We fit ordered logistic regressions [29] for simultaneously testing the influence of educational background, age and sex as predictors on the sum of present categories within each conception as response variable (function *polr*, R-package *MASS*). In addition, we included the question as factorial predictor within the model, to account for possible differences in conception expression between a basic (A) and a complex (B) question. To simultaneously compare educational backgrounds (for instance 6^th^ graders versus 10^th^ graders) we implemented pre-defined model contrast by means of function *glht (*R-package *multcomp)*, which automatically adjusts p-values for multiple testing [30]. Second, we used binomial linear models (function *glm*, R package stats) [31] for testing the influence of educational background, age and sex as predictors on the coexistence of scientific or alternative conceptions (conceptions coexist = 1, conceptions do not coexist = 0) as response variable. Again, pre-defined model contrast with automatically adjusted p-values was used to compare educational backgrounds.

## Results

By answering the open questions, the participants (n = 885) provided several concepts per question leading to a total of 1424 concepts for question A and 949 for question B, including both scientific and alternative ones. Inter- and intra-rater reliability depicted the categorisation of both questions as reliable reflected by a strength of agreement as “almost perfect” (ranging from 0.81–1) [25]. Cohen´s kappa coefficient for our questions was: Question A k = 0.97, question B k = 0.96 (inter-rater) and k = 0.98 (intra-rater) for both questions [24].

Scientific concepts in question A were that trees assimilate minerals (i.e. all inorganic substances that trees may absorb from soil), CO_2_, O_2_, light respectively sunshine and H_2_O. Alternative concepts were that trees assimilate nutrients (i.e. long-chain hydrocarbons, fats and proteins) from their environments (see S2 Table for more categories and anchor examples).

In question A, 0.5% of students reported having no idea while 6.1% did not provide any statement. In question B, 6.7% of the students had no idea and 24.0% did not provide any statement. Excluding these missing answers we received five categories of scientific (77.2%) and six categories of alternative conceptions (22.8%) for question A. Six categories of scientific conceptions (50.1%) and five categories of alternative conceptions (49.1%) were present in question B.

### Determinants of the expression of scientific and alternative conceptions

We revealed educational background as the major determinant of conception expression. In question A, freshmen of natural science and freshmen of other academic studies expressed significantly more scientific conceptions than 6^th^ and 10^th^ graders (p < .001 for all combinations) but did not differ significantly from each other (Table 1). 10^th^ graders provided significantly more scientific and alternative conceptions than 6^th^ graders. Natural science students provided significantly fewer alternative conceptions than 10^th^ graders (p < .001) and students of other academic studies (p = .01).

**Table 1:** Effect of educational background on expression of scientific and alternative conceptions tested with ordered logistic regressions and pre-defined model contrast for multiple comparisons among educational backgrounds (n = 885).

		Question A				Question B			
		Estimate	SD±	t-value	p-value^a^	Estimate	SD±	t-value	p-value^a^
**Scientific Conceptions**	6^th^ graders-10^th^ graders	-0.43	0.14	-3.11	**0.01**	-0.52	0.26	-2.04	0.16
	6^th^ graders-Other studies	-1.18	0.17	-6.83	**<0.001**	-1.22	0.31	-3.88	**<0.001**
	10^th^ graders-Other studies	-0.75	0.15	-5.01	**<0.001**	-0.70	0.27	-2.60	**0.04**
	Natural science-6^th^ graders	1.25	0.18	7.07	**<0.001**	1.90	0.32	5.93	**<0.001**
	Natural science-10^th^ graders	0.81	0.15	5.34	**<0.001**	1.38	0.27	5.06	**<0.001**
	Natural science-Other studies	0.06	0.09	0.75	0.87	0.68	0.16	4.37	**<0.001**
	Sex [male—female]	0.12	0.07	1.74	0.29	0.21	0.13	1.60	0.38
	Age	-0.11	0.02	-5.90	**<0.001**	-0.09	0.03	-2.70	**0.03**
**Alternative Conceptions**	6^th^ graders-10^th^ graders	-0.66	0.26	-2.50	**0.05**	0.02	0.24	0.10	1.00
	6^th^ graders-Other studies	-0.19	0.32	-0.60	0.93	0.30	0.32	0.94	0.77
	10th graders-Other studies	0.47	0.28	1.70	0.31	0.27	0.26	1.03	0.72
	Natural science-6^th^ graders	-0.31	0.33	-0.94	0.77	-0.77	0.33	-2.36	0.08
	Natural science-10^th^ graders	-0.97	0.28	-3.41	**<0.001**	-0.74	0.27	-2.71	**0.03**
	Natural science-Other studies	-0.50	0.16	-3.13	**0.01**	-0.47	0.16	-2.85	**0.02**
	Sex [male-female]	-0.32	0.13	-2.41	0.07	-0.14	0.13	-1.08	0.73
	Age	-0.07	0.03	-2.08	0.15	-0.02	0.03	-0.73	0.92

^a^ significant p-values are marked bold

In question B, natural science students displayed significant more scientific conceptions than all other groups (Table 1). Freshmen from other academic fields displayed significantly more scientific conceptions than 10^th^ and 6^th^ graders, whereas the latter groups did not differ significantly from one another. Natural science students expressed significantly fewer alternative conceptions than 10^th^ graders and students of other academic studies.

Students’ age yielded a significant negative effect on the expression of scientific conceptions in both questions but no effect on the expression of alternative conceptions (Table 1). We found no significant effect of sex on the expression of scientific and alternative conceptions in any model (Table 1) with one exception in Question B, where female natural science students provided significantly (p = 0.03) more alternative conception (in sum) than males (S3 Table).

### Determinants of co-existence of conception

**Fig 1:**
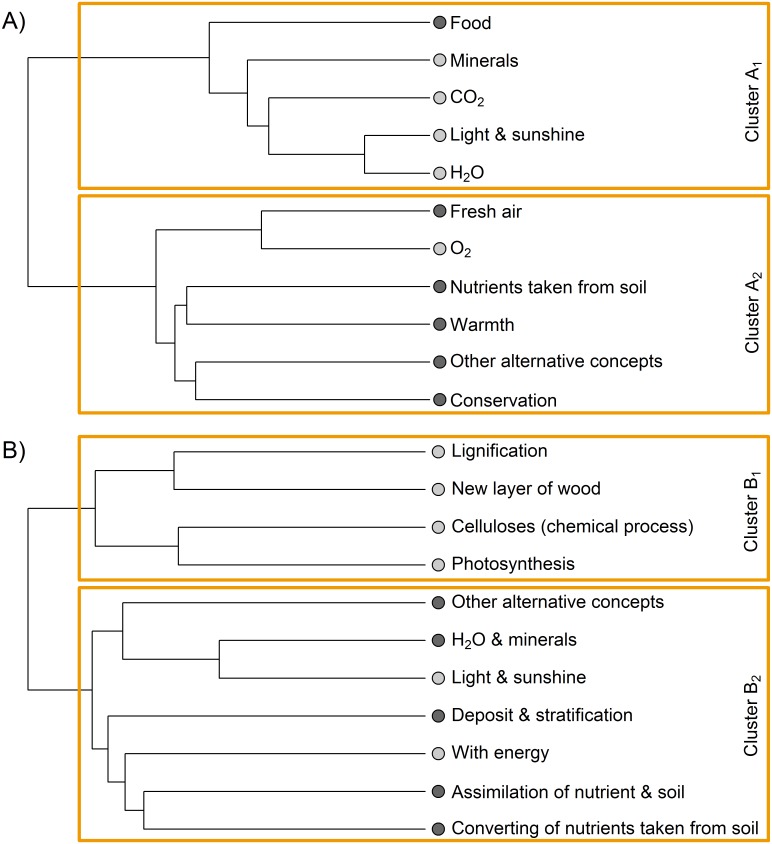
Cluster analysis (based on ward´s method and k-mean procedure) for co-existence of scientific (light grey) and alternative (dark grey) conceptions (N = 885).

Both cluster analysis approaches revealed congruently two clear clusters in both questions (Fig 1). Ward´s method and k-mean procedure for question A yielded a coefficient of contingency of *C* = .82 (with *C*_max_ = .83, n = 885 p < .001) whereas question B had a coefficient of contingency of *C* = .50 (with *C*_max_ = .83, n = 885, p < .001). In question A one alternative conception “food” and all scientific conceptions (“Minerals”, “CO_2_,” “Light & sunshine” and “H_2_O”) except one were assigned to cluster 1. Cluster 2 consisted of one scientific conception (“O_2_”) and all alternative conceptions (“Fresh air”, “Nutrients taken from soil”, “Warmth”, “Other alternative concepts” and “Conservation”) except one. For question B four out of six scientific conceptions (“Lignification”, “New layer of wood”, “Celluloses (chemical process)”, and “Photosynthesis”) were found in cluster 1 while all alternative conceptions (“Other alternative concepts”, “H_2_O & minerals”, “Deposit and stratification”, “Assimilation of nutrient and soil” plus “Converting of nutrients taken from soil”,) mixed with two scientific conceptions (“Light & sunshine” and “With energy”) were located in cluster 2 (Fig 1).

Older students provided significantly fewer fragmented conceptions than younger students in question A. 6^th^ graders provided significantly more co-existing conceptions than 10^th^ graders and students of other academic studies. Natural science students displayed a more fragmented knowledge profile than students from other studies. Female students had more co-existing conceptions than male students in question A. However, we found no significant effects of educational background or age within question B (Table 2).

**Table 2:** Co-existence of scientific and alternative conceptions in dependence of educational background (6^th^ graders, 10^th^ graders, natural science freshmen, and other academia studies freshmen), sex and age; based on binomial-linear models and pre-defined model contrast for multiple comparisons among educational backgrounds (n = 885).

	Question A				Question B			
	Estimate	± SD	t-value	p-value^a^	Estimate	± SD	t-value	p-value^a^
Sex [male—female]	-0.28	0.14	-1.99	**0.05**	-0.07	0.16	-0.47	0.64
Age	-0.12	0.04	-3.48	**0.00**	-0.03	0.04	-0.75	0.45
10^th^ graders—Other studies	0.24	0.32	0.74	0.88	-0.10	0.32	-0.31	0.99
6t^h^ graders—Other studies	-0.94	0.34	-2.74	**0.03**	-0.35	0.37	-0.95	0.77
Natural science—Other studies	-0.49	0.17	-2.95	**0.02**	-0.17	0.19	-0.93	0.78
6^th^ graders—10^th^ graders	-1.18	0.30	-3.89	**<0.001**	-0.25	0.30	-0.84	0.83
Natural science—10^th^ graders	-0.73	0.32	-2.26	0.10	-0.07	0.32	-0.23	1.00
Natural science—6^th^ graders	0.45	0.35	1.29	0.55	0.18	0.38	0.47	0.96

^a^adjusted p values reported (single-step method), significant p-values marked bold

### Different presence of scientific and alternative conceptions within the two questions

**Fig 2:**
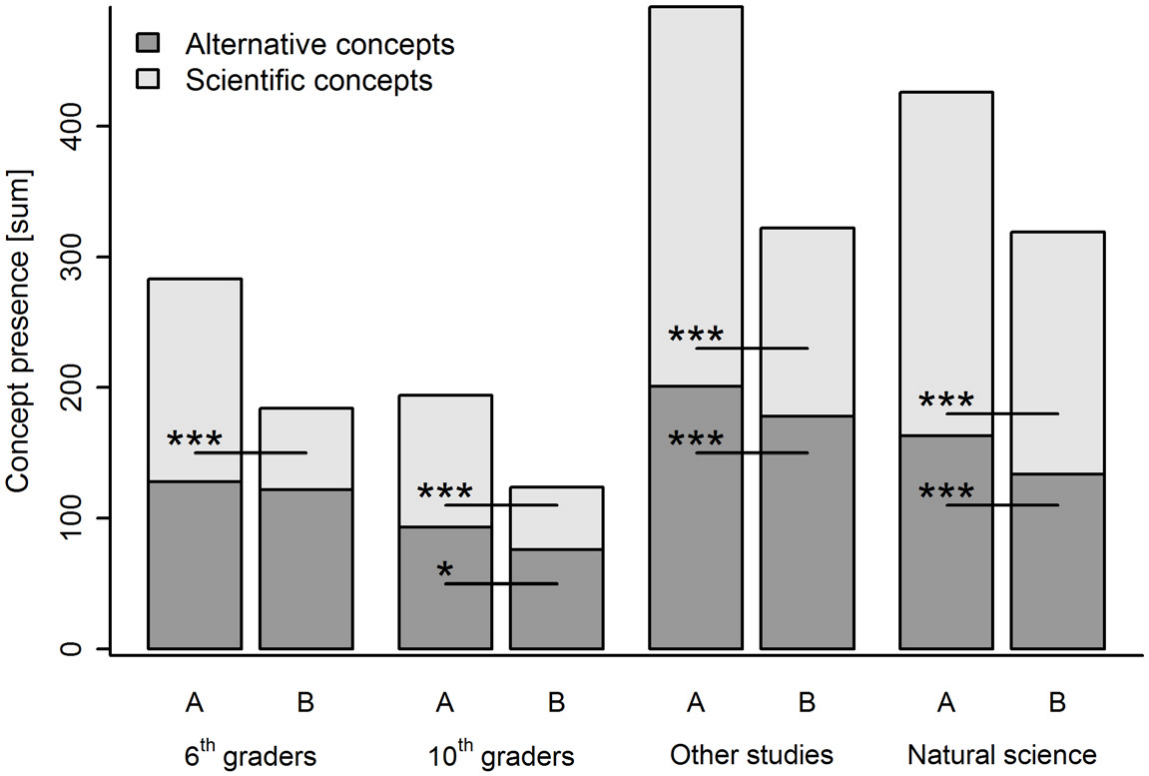
Presence of scientific and alternative conceptions divided by questions and educational backgrounds (N = 885, based on GLM, concepts as command variable with educational background, sex and age as random factor, for multiple comparison adjusted significance levels are marked by *<0.05, ** <0.01, *** <0.001 above lines, for exact p-values see S4 Table).

Independent of educational background, significantly more scientific conceptions were present in question A compared to question B (S4 Table). Freshmen of natural sciences and other academic studies displayed significantly more alternative conceptions in question A than in question B. 10^th^ graders provided more alternative conceptions in A than in B while 6^th^ graders showed no significant difference in the expression of alternative conceptions (Fig 2 and S4 Table).

## Discussion

Our study demonstrated that scientific and alternative conceptions can co-exist in the framework of tree assimilation, photosynthesis and wood synthesis. Furthermore, older students and students with advanced educational background expressed fewer alternative conceptions, resulting in a more integrated knowledge profile, which is in line with previous studies. For example Liu and Lesniak [32] demonstrated that students’ conceptions of composition of substances integrates from “macroscopic to microscopic” from 1^st^ to 10^th^ grade. Thus, older and higher educated students provide more microscopic explanation.

### Education fosters accumulation of scientific conceptions

Bledsoe [33] explained a “learning sequence” in which decreasing alternative conceptions occur in as students’ understanding develops. Therefore, we might expect an increase in scientific conceptions parallel to age and education. This seems true for question A as age was a determining factor and more scientific conceptions were found with increasing educational levels except for both freshman populations. An explanation for the missing differences between natural science freshman and other academic studies freshman might originate in the phenomenon called plant blindness [34]. Schussler and Olzak [35] showed that even college students enrolled in botany classes exhibit this phenomenon of ignoring plants within the individual concept architecture. Since the 1980s teachers have been aware of the difficulty of teaching photosynthesis, and therefore they regard photosynthesis as the most important topic [36]. Due to difficulty and importance the topic is constantly implemented within 6^th^ and 10^th^ grades as well as senior classes and higher education syllabi. Therefore, repeated attention to the topic might lead to a higher expectation for the integration of scientific conceptions following increasing educational levels and age, respectively. Our data suggest a similar pattern as natural science students provided significantly more scientific conceptions than all the other groups, while freshmen from other academic fields expressed significantly more scientific conceptions than 6^th^ and 10^th^ graders.

Concrete reasons for the different patterns should be investigated further in more qualitative studies using interviews. However, we can propose some reasons based on previous research.

Finley and colleagues demonstrated knowledge to be forgotten when it was gained due to pure memorising efforts made to properly pass exams [36]. Ekici and colleagues reason students to be memorising the chemical equation of photosynthesis without understanding the underlying biological principles [37]. Both studies suggest a possible explanation for the similar scoring of our 6^th^ and 10^th^ graders who did not differ significantly within the expression of scientific conceptions. The groups are likely to have forgotten their old knowledge about photosynthesis which they learnt in the 6^th^ grade as it was not needed until 10^th^ which apparently yielded a lack of engagement with the topic that could have formed a deeper understanding.

Our university freshmen, however, were confronted more often with the correct scientific understanding of photosynthesis during their educational life leading to the integration of more scientific conceptions. As expected, natural science students provided more specific and detailed information, for example including enzymes within the process of photosynthesis and wood synthesis, but like all other groups often lacked a meaningful and general view which is in line with existing literature [37–39].

We could not find any significant effect of sex on expression of conceptions, except that female natural science students provided a higher number of alternative conceptions than males (S3 Table). The literature reports gender differences in language use “in form, topic, content and function” [37] (p.116): Females often tend to use more words implying their opinion, “while men tend to orientate to its referential function” [38] (p.30). That indicates why in our case, we found females to enumerate more alternative concepts than males only in that special case. “In sum scores, boys had a larger score variance in total general knowledge and most domains, with exceptions in […] Biology […] where girls had a larger score variance” [40]. Tran and colleagues concluded that differences in knowledge of biology between sexes are inconsistent while overall there was no evidence for biologically differentiated interests between female and male students. They also suggested that “previous research likely overestimated sex differences in general knowledge” [40].

### Fragmented knowledge profiles: “Trees live on soil and sunshine!”

Despite our educational subgroups being increasingly taught scientific information, the presence of alternative conceptions remained almost untouched. This discrepancy is in agreement with Sinatra et al. [41] who depicted naïve positions as coexisting with scientific understanding. This phenomenon results from the nature of alternative conception which are regarded as being resistant to change and thus difficult to overcome by traditional methods [42]. The majority of study participants held scientifically correct concepts about substances which a tree needs to assimilate from its environment (question A), but these occurred along with alternative ones, as the individual statement ‘trees live on soil and sunshine!’ highlights. The correct scientific conception “sunshine” (needed for photosynthesis) is nested within one cluster (A1, question A) together with the alternative conception “food” and other scientific conceptions. Consequently, a fragmented knowledge profile exists which is congruent with our Cluster A2 (question A) as it comprises the scientific conception “O_2_” in parallel with the alternative conception “nutrients taken from soil”. The alternative concept that “food is needed to grow” is a well-known alternative conception within literature [20,22,33,38]. Students tend to see plants as dependent on humans and even as inferior [38]. Based on their experience “food” is needed for human and animal life, which probably leads to the conception of plants being dependent on “food” from the environment [22] which especially the younger students refer to as “food” [33]. Even 8^th^ graders often state that “plants get their food from their environment as animals do” [35] (p.115). Students mentioned oxygen which is needed for respiration and energy generation to power photosynthesis as an endergonic process. Interestingly, younger students seemingly do not differentiate between “O_2_” and “fresh air” as these two conceptions occur within one single clade. However, whether oxygen was mentioned due to humanisation or to other reasons cannot be distinguished. Probably the first aspect was on hand in most cases as will be concluded when taking question B into account later. The alternative conception “conservation” in question A emphasises students’ assumption about plants relying on man. “Warmth” and “fresh air” presumably testify to analogies which students drew from their own experiences by transferring these views to plant life.

Regarding question B the first cluster (B1) can be described as a “scientific” cluster, consisting of sophisticated concepts which lead to a correct explanation of wood synthesis. This cluster was dominantly but not exclusively provided by natural scientists. Cluster B2 can be described as a “fragmented cluster” which comprises a mixture of scientific and alternative conceptions. Beyond two scientific conceptions and four other alternative conceptions, “assimilation of nutrition and soil”, a prominent alternative conception, is part of this cluster. This conception is well-known in the literature. Students of the “fragmented” cluster apparently are not able to approach the biological topic of plants on a more chemical basis, although energy is specifically mentioned as a concept. This is in line with Stavy et al. [38] who accounts for students’ difficulty explaining biological phenomena from a chemical perspective as “students try to construct a coherent and logical […] view of the world from limited knowledge they possess” (p.110) about photosynthesis and related processes. It appears that students tend to reorganise their knowledge only within one domain but not across different fields. Additionally our study demonstrated that older students had less fragmented knowledge than younger, meaning less co-existence of alternative and scientific conceptions. Interestingly, in question A, natural science students had a higher co-existence of the two different conceptions than other academic studies (Table 2). One reason probably is that natural scientists enumerated all scientific correct answers they knew due to their education but additionally provided all alternative concepts they ever had. In question B, we detected no differences between the educational backgrounds. This finding suggests that alternative conceptions are very hard to overcome [42] and instead of replacing alternative conceptions with scientifically correct ones, students keep both. Our results as well as the existing literature support the coexistence of different conceptions. This process of generating knowledge, meaning learning scientifically correct concepts whilst keeping the alternative ones, was previously described by Vosniadou and Ortony [43]. All subgroups featured uniform alternative conceptions probably because these “worked” in their everyday lives as described by Bledsoe [33]. This is in line with Schneider and Hardy [7] who described “clear evidence for the coexistence of inconsistent pieces of knowledge in learners” (p.1647), which is confirmed by our findings. These co-existing conceptions highlight the need to support students in reorganising their accumulated knowledge.

### Complexity fosters understanding

All students, irrespective of educational background, provided significantly more scientific conceptions in question A than in question B. This is possibly caused by the complexity of question B, which focused on a deeper understanding of the biological wood synthesis process in contrast to the more basic question A which focused on enumerating factors of tree assimilation. The two freshman populations and the participating 10^th^ graders provided significantly more alternative conceptions in question A than in B while we did not find any significant difference within 6^th^ graders. One reason could be the varying complexity of both questions: whereas question A requires enumeration of important substances, question B needs a deeper understanding. Consequently, older students may have named just everything they knew in question A while in question B they tended to reject any answer or provided the statement “I don’t know”. In contrast, our sampled 6^th^ graders did not hesitate to creatively explain their understanding of wood synthesis using alternative conceptions as they had in question A.

### Photosynthesis challenges biological education

Difficulties in understanding photo-autotrophy have been known since the 1980s [38] revealing, amongst others, the alternative conception “nutrition taken from soil”. Students still express a need for “food” from soil in connection with photosynthesis nowadays, despite numerous classroom efforts in the past. Carlson [39] describes teachers as teaching according to their own conceptual understanding which can differ from a scientifically correct one. Thus, students’ alternative conceptions just echo a teacher’s understanding [44]. As students of the 1980s nowadays are likely to serve as in-service teachers, alternative conceptions of the 1980s can still be taught to the next generations. Hence further supporting mechanisms in teacher education are needed to restructure knowledge for appropriate teaching, particularly since even natural science freshmen displayed co-existence of both conception levels. However, repeatedly encountering photosynthesis during their education increased the expression of scientific conceptions by our participants, reflected in increasing expression of scientific conceptions among higher educational levels (Table 1). Nevertheless, daily life does not require understanding the complete interrelations of this thematic field. As Bledsoe [33] explains “knowing some elementary ideas” (p.31) is sufficient. It is stated that “[…] naïve theories survive the acquisition of a mutually incompatible scientific theory, coexisting with that theory for many years to follow.” [43] (p. 209). Consequently there seems to be no need to reorganise existing knowledge structures even for natural scientists. Against this background it is crucial to see if scientists who are working in the field of photosynthesis still hold some alternative conceptions. As Shtulman and Valcarel [45] found, people experienced in science and under time-pressure are slow to verify naïve statements. On the other hand, Masson et al. [46] detected neurological reasons for the inhibition of alternative conceptions by scientific experts. Thus Sinatra et al. [41] highlights that even well-trained scientists are not immune to hold alternative conceptions. Consequently, student-tailored interventions are needed to promote a conceptual change within our sample. Effective ways are student-centred, hands-on experiences or concept cartoons which confront students directly with their alternative conceptions [7,22,37,47]. Providing further support in a “real-world context” would be promising as well as student’s conceptions are context- and even situation-specific [48].

## Conclusions

Despite significant research effort to improve teaching strategies has been undertaken since the 1980s, scientific and alternative conceptions still co-exist in students´ minds. Throughout our analysis, educational background was the most important determinant for increasing scientific conceptions and fosters accumulation of scientific concepts. Nevertheless, even science students kept some alternative conceptions although they knew the correct scientific ones. Hence our data suggest that teaching of photosynthesis and wood assimilation should be repeated along ascending educational levels to foster understanding and overcome alternative conceptions. Such repeated teaching should not only take place in classrooms, but also in university courses to strengthen future teachers’ scientific conceptions, which will then be transferred to learners.

## Supporting Information

S1 Table Sample description class-divided according to educational background (n = 885). (DOCX)

S2 Table Categories per level of conception within question A and B. (DOCX)

S3 Table Sex Effect on scientific and alternative concepts. (DOCX)

S4 Table Conception levels in comparison between question A and B. (DOCX)
