# Supplementary material for: “Trees Live on Soil and Sunshine!”- Coexistence of Scientific and Alternative Conception of Tree Assimilation
Source: PLoS One. 2016 Jan 25;11(1):e0147802. doi: 10.1371/journal.pone.0147802 (PMC4725716; doi:10.1371/journal.pone.0147802)
Supplement: S1 Table — (DOCX) [file pone.0147802.s001.docx]

S1 Table: Sample description class-divided according to educational background (N=885).

| Educational background | Students | Male [%] | Female [%] | Age ± SD |
| --- | --- | --- | --- | --- |
| 6th grade | 167 | 47.9 | 52.1 | 12.86±0.98 |
| 10th grade | 103 | 51.5 | 48.5 | 15.60±0.60 |
| Other studies | 309 | 35.2 | 64.8 | 20.92±2.60 |
| Natural science | 306 | 56.1 | 43.9 | 20.59±2.00 |
| Total | 885 | 46.2 | 53.8 | 18.71±3.87 |
