# Supplementary material for: “Trees Live on Soil and Sunshine!”- Coexistence of Scientific and Alternative Conception of Tree Assimilation
Source: PLoS One. 2016 Jan 25;11(1):e0147802. doi: 10.1371/journal.pone.0147802 (PMC4725716; doi:10.1371/journal.pone.0147802)
Supplement: S2 Table — (DOCX) [file pone.0147802.s002.docx]

**S2 Table:** Categories per level of conception within question A and B.

| ***Level of Conception*** | **Question A** | **Example A** | **Question B** | **Example B** |
| --- | --- | --- | --- | --- |
| *Scientific Conception* | H_2_O | “The oak needs to take water from the soil.”(female, age 13)^1^ | Photo-synthesis | “By means of sunlight and CO2 the tree conducts photosynthesis [...] thereby it thrives and prospers.” (male, age 25)^4^ |
|  | Light & sunshine | “It needs to assimilate [...], light to become that big.” (male, age 13)^1^ | Celluloses (chemical process) | “The wood of the trunk derives of a chemical process in which cellulose is produced”(male, age 19)^4^ |
|  | CO_2_ | “Using photosynthesis the tree assimilates carbon dioxide [...]” (male, age 15)^2^ | Lignification | “[...] Lignin is brought into the outer cells, these cells become lignified.” (female, age 18)^4^ |
|  | O_2_ | “It needs to take in oxygen [...] to become that big”(male, age 12)^1^ | With energy | “...energy is used to foster cell growth and cleavage...”(male, 20)^3^ |
|  | Minerals | “To survive so many years the tree needs to assimilate water solute mineral nutrients [...]”  (female, age 13) ^2^ | Sun & light | “using sunlight the tree grows upwards and the trunk gets thickened”(female, age 15)^2^ |
|  |  |  | New layer of wood | “A tree contains annual rings inside. Each year a new ring is formed and thus the tree grows.”(male, age 14)^2^ |
| *Alternative conception* | Fresh air | “…needs fresh air, otherwise it dies” (male, age 20)^3^ | Converting of nutrients | “It converts nutrients into cells...”(female, age 21)^3^ |
|  | Food (“nutrients”) | “the tree needs to assimilate [...], nutrients [...].“(female, age 15)^2^ | Deposit & stratification | Components are deposited inside and sometimes more space is needed therefore the tree gets thicker.”(male, age 15)^2^ |
|  | Warmth | “sun and the adequate warmth are important”(male, age 12)^1^ | H_2_O & minerals | “First, there is a small tree then it grows because of water and mineral nutrients...”(male, age 12)^1^ |
|  | Conservation | “This tree needs conservation.”(female, age 14)^2^ |  |  |
|  | Nutrient (taken) from soil | “It needs to assimilate important nutrients from the soil”(male, age 12)^1^ | Assimilation of nutrient & soil | “The tree assimilates nutrients and soil and by utilising these it produces wood.”(male, age 19)^3^ |
|  | Other alternative conception | “...and love <3”(female, age 20)^3^ | Other alternative conception | “if the tree is happy it grows better”(female, age 14)^2^ |
| *No idea* | No idea | “ No idea I’m not a “tree researcher””(male age 14)^2^ | No idea | “I don’t know!” (female, age 13)^1^ |
| *No answer* | No answer | / | No answer | / |

^1^6^th^ grade, ^2^10^th^ grade, ^3^freshman other studies, ^4^freshman natural science, N=885, generated with an inductive bottom-up approach after Mayring (2001)
