# Supplementary material for: “Trees Live on Soil and Sunshine!”- Coexistence of Scientific and Alternative Conception of Tree Assimilation
Source: PLoS One. 2016 Jan 25;11(1):e0147802. doi: 10.1371/journal.pone.0147802 (PMC4725716; doi:10.1371/journal.pone.0147802)
Supplement: S3 Table — (DOCX) [file pone.0147802.s003.docx]

**S3 Table:** Sex Effect on scientific and alternative conceptions.

|  |  |  | **Estimate** | **± SD** | **t-value** | **p-value^a^** |  |
| --- | --- | --- | --- | --- | --- | --- | --- |
| **Question A** | **Scientific Conception** | overall sex female - male | 0.12 | 0.25 | 0.47 | 0.64 |  |
|  |  | 6th graders female - male | 0.11 | 0.60 | 0.19 | 1.00 |  |
|  |  | 10th graders female - male | 14.29 | 557.81 | 0.03 | 1.00 |  |
|  |  | Other studies female - male | -0.02 | 0.56 | -0.03 | 1.00 |  |
|  |  | Natural science female - male | 0.00 | 0.37 | 0.01 | 1.00 |  |
|  |  | age | **-0.21** | **0.03** | **-6.21** | **<0.001** | *** |
|  | **Alternative Conception** | overall sex female - male | -0.15 | 0.14 | -1.08 | 0.28 |  |
|  |  | 6th graders female - male | 0.14 | 0.32 | 0.43 | 0.99 |  |
|  |  | 10th graders female - male | 0.28 | 0.49 | 0.57 | 0.97 |  |
|  |  | Other studies female - male | 0.50 | 0.24 | 2.10 | 0.14 |  |
|  |  | Natural science female - male | 0.04 | 0.24 | 0.15 | 1.00 |  |
|  |  | age | **-0.08** | **0.02** | **-4.34** | **<0.001** | *** |
| **Question B** | **Scientific Conception** | overall sex female - male | 0.03 | 0.14 | 0.19 | 0.85 |  |
|  |  | 6th graders female - male | 0.08 | 0.32 | 0.25 | 1.00 |  |
|  |  | 10th graders female - male | -0.37 | 0.40 | -0.93 | 0.83 |  |
|  |  | Other studies female - male | -0.36 | 0.23 | -1.53 | 0.42 |  |
|  |  | Natural science female - male | 0.11 | 0.24 | 0.47 | 0.98 |  |
|  |  | age | **0.03** | **0.02** | **1.93** | **0.05** | * |
|  | **Alternative Conception** | overall sex female - male | -0.15 | 0.14 | -1.07 | 0.28 |  |
|  |  | 6th graders female - male | -0.05 | 0.31 | -0.16 | 1.00 |  |
|  |  | 10th graders female - male | 0.13 | 0.40 | 0.32 | 1.00 |  |
|  |  | Other studies female - male | 0.05 | 0.24 | 0.21 | 1.00 |  |
|  |  | Natural science female - male | **0.76** | **0.29** | **2.67** | **0.03** | * |
|  |  | age | **-0.08** | **0.02** | **-4.24** | **<0.001** | *** |

N=885, ^a^ significant p-values marked bold, signif. codes: 0 ‘***’ 0.001 ‘**’ 0.01 ‘*’ 0.05 ‘.’ 0.1 ‘,’ 1, based on general linear model (GLM), concepts as command variable and educational background, sex and age as random factor
