# Supplementary material for: “Trees Live on Soil and Sunshine!”- Coexistence of Scientific and Alternative Conception of Tree Assimilation
Source: PLoS One. 2016 Jan 25;11(1):e0147802. doi: 10.1371/journal.pone.0147802 (PMC4725716; doi:10.1371/journal.pone.0147802)
Supplement: S4 Table — (DOCX) [file pone.0147802.s004.docx]

**S4 Table:** Conception levels in comparison between question A and B.

|  | Question A vs. question B | **Estimate** | **± SD** | **z-value** | **p-value^a^** |  |
| --- | --- | --- | --- | --- | --- | --- |
| Scientific conception | Scienctific concept overall | **-2.47** | **0.14** | **-17.41** | **<0.001** | *** |
|  | 6th graders | **-3.10** | **0.34** | **-9.12** | **<0.001** | *** |
|  | 10th graders | **-4.06** | **0.74** | **-5.49** | **<0.001** | *** |
|  | Other studies | **-3.13** | **0.29** | **-10.84** | **<0.001** | *** |
|  | Natural science | **-1.41** | **0.20** | **-6.94** | **<0.001** | *** |
| Alternative conception | Alternative concept overall | **-0.63** | **0.10** | **-6.38** | **<0.001** | *** |
|  | 6th graders | -0.38 | 0.22 | -1.67 | 0.33 |  |
|  | 10th graders | **-0.95** | **0.32** | **-3.00** | **0.01** | * |
|  | Other studies | **-0.68** | **0.16** | **-4.12** | **<0.001** | *** |
|  | Natural science | **-0.62** | **0.17** | **-3.75** | **<0.001** | *** |

N=885, ^a^ significant p-values marked bold, signif. codes: 0 ‘***’ 0.001 ‘**’ 0.01 ‘*’ 0.05 ‘.’ 0.1 ‘,’ 1, based on GLM (general linear model), concepts as command variable with educational background, sex and age as random factor
